# The role of Src kinase in the biology and pathogenesis of *Acanthamoeba castellanii*

**DOI:** 10.1186/1756-3305-5-112

**Published:** 2012-06-07

**Authors:** Ruqaiyyah Siddiqui, Junaid Iqbal, Marie-josée Maugueret, Naveed Ahmed Khan

**Affiliations:** 1 Department of Biological and Biomedical Sciences, The Aga Khan University, Karachi, Pakistan; 2 School of Biological and Chemical Sciences, University of London, Birkbeck, UK

**Keywords:** *Acanthamoeba*, Pathogenesis, Encephalitis, Src kinase

## Abstract

**Background:**

*Acanthamoeba* species are the causative agents of fatal granulomatous encephalitis in humans. Haematogenous spread is thought to be a primary step, followed by blood–brain barrier penetration, in the transmission of *Acanthmaoeba* into the central nervous system, but the associated molecular mechanisms remain unclear. Here, we evaluated the role of Src, a non-receptor protein tyrosine kinase in the biology and pathogenesis of *Acanthamoeba*.

**Methods:**

Amoebistatic and amoebicidal assays were performed by incubating amoeba in the presence of Src kinase-selective inhibitor, PP2 (4-amino-5-(4-chlorophenyl)-7-(*t*-butyl)pyrazolo[3,4-*d*]pyrimidine) and its inactive analog, PP3 (4-amino-7-phenylpyrazolo[3,4-*d*]pyrimidine). Using this inhibitor, the role of Src kinase in *A. castellanii* interactions with *Escherichia coli* was determined. Zymographic assays were performed to study effects of Src kinase on extracellular proteolytic activities of *A. castellanii*. The human brain microvascular endothelial cells were used to determine the effects of Src kinase on *A. castellanii* adhesion to and cytotoxicity of host cells.

**Results:**

Inhibition of Src kinase using a specific inhibitor, PP2 (4-amino-5-(4 chlorophenyl)-7-(*t*-butyl)pyrazolo [3,4-*d*] pyrimidine) but not its inactive analog, PP3 (4-amino-7-phenylpyrazolo[3,4-*d*] pyrimidine), had detrimental effects on the growth of *A. castellanii* (keratitis isolate, belonging to the T4 genotype). Interestingly, inhibition of Src kinase hampered the phagocytic ability of *A. castellanii*, as measured by the uptake of non-invasive bacteria, but, on the contrary, invasion by pathogenic bacteria was enhanced. Zymographic assays revealed that inhibition of Src kinases reduced extracellular protease activities of *A. castellanii*. Src kinase inhibition had no significant effect on *A. castellanii* binding to and cytotoxicity of primary human brain microvascular endothelial cells, which constitute the blood–brain barrier.

**Conclusions:**

For the first time, these findings demonstrated that Src kinase is involved in *A. castellanii* proliferation, protease secretions and phagocytic properties. Conversely, invasion of *Acanthamoeba* by pathogenic bacteria was stimulated by Src kinase inhibition.

## Background

Based on the ribosomal DNA (rDNA) sequencing, the genus *Acanthamoeba* represents 17 different groups, i.e., T1 – T17 [1-3]. The basis of this scheme is that each group (or genotype) exhibits ≥ 5% rDNA sequence divergence from other genotypes. Pathogenic *Acanthamoeba* (predominantly belonging to the T4 genotype) can produce painful, blinding keratitis, normally associated with contact lens use or a fatal granulomatous amoebic encephalitis (GAE), primarily associated with immunocompromised patients [4-6]. The most distressing aspect is that the prognosis is poor, despite advances in antimicrobial chemotherapy and supportive care. In particular, there is very limited success in the treatment of GAE, which is most likely due to the inability of drugs to cross the blood–brain barrier into the central nervous system (CNS) to target pathogen, non-specific toxicity, and amoebae transformation into resistant cyst forms. However, alkylphosphocholine compounds show promise [7]. Among them, hexadecylphosphocholine has been shown to possess anti-*Acanthamoeba* characteristics and has the ability to cross the blood–brain barrier. To date, their mode of action and *in vivo* efficacy are unknown. Clearly, there is a need to find novel strategies in the rational development of therapeutic interventions.

The burden of *Acanthamoeba* keratitis on human health is estimated at 0.01 – 1.5 infections per 10,000 people who wear contact lens [4]. In contrast, a true or even approximate burden of encephalitis on human health is not known. As indicated above, GAE infections are usually limited to immunocompromised patients, such as those with HIV/AIDS [8]. GAE can also occur in chronically ill or debilitated individuals, some of whom take immunosuppressive therapy or broad-spectrum antibiotics [8]. The pathogenesis of the disease is not clearly understood, although the route of infection is thought to relate to the inhalation of the amoebae through the nasal passages and lungs or infection through skin lesions [8]. The respiratory and cutaneous infections tend to last for a few months, whereas infection involving the CNS can be fatal within days [5,6]. The haematogenous spread by circulating amoebae is a prerequisite for GAE, followed by their traversal of the blood–brain barrier, but the underlying mechanisms remain incompletely understood [4,9]. It is possible that new targets may be found in the signal transduction pathways that can affect amoeba survival and host-pathogen interactions. Src is a member of a larger family of related tyrosine kinases that includes Fyn, Yes, Lck, Blk, Lyn, Hck, Yrk and Fgr. Src is a non-receptor protein tyrosine kinase and its activation is mainly regulated by phosphorylation at the tyrosine 416 residue [10]. Src signalling has been implicated in a variety of cellular processes, including cell growth, survival, cellular transformation and motility [11,12]. For the first time, in the present study, we investigated the role of Src kinase in *A. castellanii*.

## Methods

All chemicals were purchased from Sigma (Poole, Dorset, UK), unless otherwise stated.

### Culturing of *Acanthamoeba castellanii*

An *A. castellanii* isolate belonging to the T4 genotype was obtained from the American Type Culture Collection (ATCC50492), and sourced from a keratitis patient. The cells were grown axenically in 10 ml of PYG medium [0.75% (w/v) proteose peptone, 0.75% (w/v) yeast extract and 1.5% (w/v) glucose] (Oxoid Ltd., Basingstoke, UK) in a T-75 tissue culture flask at 30 °C as previously described [13]. The medium was refreshed 17 – 20 h prior to experiments, which resulted in > 95% of amoebae in the trophozoite form.

### Human brain microvascular endothelial cell (HBMEC) culture

The primary BMEC were isolated from the human tissue and purified by fluorescent activated cell sorting (FACS) and exhibited endothelial characteristics, such as expression of endothelial markers, F-VIII, carbonic anhydrase IV and uptake of acetylated low density lipoprotein (AcLDL) as previously described [13,14]. HBMEC were grown in RPMI-1640 containing 10% foetal bovine serum, 10% NuSerum, 2 mM glutamine, 1 mM pyruvate, penicillin (100U/ml), streptomycin (100U/ml), non-essential amino acids and vitamins (Invitrogen, Paisley, UK) [13,14].

### Amoebistatic and amoebicidal assays

*A. castellanii* were grown to confluency in 24-well plates. Next day, plates were washed with PBS to remove unbound amoebae. The varying concentrations of a potent, Src kinase-selective inhibitor, PP2 (4-amino-5-(4-chlorophenyl)-7-(*t*-butyl)pyrazolo[3,4-*d*pyrimidine) [15] and its inactive analog, PP3 (4-amino-7-phenylpyrazolo[3,4-*d*pyrimidine) [16] (Calbiochem, San Diego, CA, USA) were added. For growth assays, amoebae plus inhibitors were incubated in PYG medium for various intervals of time, followed by haemocytometer counting. For viability assays, amoeba plus inhibitors were incubated in PBS for 24 h, and viability determined by Trypan blue exclusion testing using haemocytometer counting [17]. For controls, normal growth rates of *A. castellanii* were determined using growth medium alone, i.e., PYG or amoebae incubated in PBS in the absence of inhibitors.

### Zymographic assays

The extracellular proteolytic activities of *Acanthamoeba* were determined using zymographic assays as previously described [18]. Briefly, *A. castellanii* were incubated in the presence or absence of various concentrations of Src kinase inhibitors for 24 h. Next day, cell-free supernatants (CM, conditioned medium) were collected by centrifugation. The CM were electrophoresed on sodium dodecyl sulfate-polyacrylamide gel electrophoresis (SDS-PAGE) containing gelatin (2 mg/ml) as a protease substrate. After electrophoresis, gels were washed in 2.5% Triton X-100 (wt/vol) for 60 min, then incubated in developing buffer (50 mM Tris–HCl, pH 7.5, containing 10 mM CaCl_2_) at 37 °C overnight. Next day, gels were stained with Coomassie Brilliant Blue. Areas of gelatin digestion were visualised as non-staining regions in the gel.

### Phagocytosis assays using live, non-invasive *E. coli* K-12

*A. castellanii* were grown to confluency in 24-well plates. Next day, plates were washed with PBS to remove unbound amoebae. *A. castellanii* were incubated with various concentrations of Src kinase inhibitor, PP2 and its inactive analog, PP3 in RPMI at room temperature for 30 min. After this incubation, *A. castellanii* were washed with PBS to remove any residual inhibitor. Next, live *Escherichia coli* K-12 (10^7^/well), a non-invasive laboratory strain, HB101 were added and plates were incubated for 45 min to allow phagocytic uptake. Following this incubation, supernatants were removed and gentamicin was added (final conc. 100 μg/ml for 45 min) to kill any remaining extracellular *E. coli*. Next, the numbers of *A. castellanii* were determined using haemocytometer counting. Finally, *A. castellanii* were solubilized with 0.5% SDS and *E. coli* counts were determined by inoculating lysates on nutrient agar plates. This allowed the determination of any intracellular *E. coli*.

### Determination of relative phagocytic activity

The level of *A. castellanii* phagocytosis was determined as follows: No. of *E. coli* colony forming units (cfu)/Total number of *A. castellanii* x 100 = % phagocytosis. Results are expressed as relative phagocytosis (% phagocytosis in untreated *A. castellanii* was considered as 100% and levels of phagocytosis in inhibitor-treated *A. castellanii* are shown as percentage change).

### Invasion assays using live pathogenic E. coli K1

Invasion assays were performed as for phagocytosis assays except that an invasive strain of *E. coli* K1, RS218 (O18:K1:H7) a cerebrospinal fluid isolate from a meningitis patient) was used as opposed to the non-invasive K-12.

### Adhesion assays

To determine the ability of *A. castellanii* to bind HBMEC, adhesion assays were performed [19]. Briefly, HBMEC were grown to confluency in 24-well plates. *A. castellanii* (4 x 10^5^ amoebae/well) were pre-incubated with various concentrations of inhibitor for 30 min. After this incubation, *A. castellanii* were washed with PBS to remove residual inhibitor. Finally, *A. castellanii* were added to HBMEC monolayers in RPMI 1640 containing 2 mM glutamine, 1 mM pyruvate and non-essential amino acids) and plates incubated at 37 °C in a 5% CO_2_ incubator. After 1 h incubation, the unbound amoebae were counted using a haemocytometer and the numbers of bound amoebae were calculated as follows: No. of unbound amoebae/Total number of amoebae x 100 = % unbound amoebae. The numbers of bound amoebae were deduced as follows: % unbound amoebae – 100 = % bound amoebae.

### Cytotoxicity assays

Cytotoxicity assays were performed as previously described [19]. Briefly, *A. castellanii* were pre-treated with PP2 and PP3 and incubated with HBMEC monolayers grown in 24-well plates as described for adhesion assays. Plates were incubated at 37 °C in a 5% CO_2_ incubator and periodically observed for cytopathic effects for up to 24 h. At the end of this incubation period, supernatants were collected and cytotoxicity was determined by measuring lactate dehydrogenase (LDH) release (cytotoxicity detection kit; Roche Applied Science, Lewes, East Sussex, UK). Briefly, conditioned media of co-cultures of *A. castellanii* and HBMEC were collected and percentage LDH was detected as follows: (sample value – control value/total LDH release – control value x 100 = % cytotoxicity). Control values were obtained from HBMEC incubated in RPMI alone. Total LDH release was determined from HBMEC treated with 1% Triton X-100 for 30 min at 37 °C.

### *Acanthamoeba* Src kinase identification and sequence alignments

Putative *Acanthamoeba* Src kinase protein was searched in *Acanthamoeba castellanii* genome database, hosted by The Human Genome Sequencing Center, Baylor College of Medicine (http://blast.hgsc.bcm.tmc.edu/blast.hgsc?organism = AcastellaniNeff), using closely related *Monosiga brevicollis* Src1 kinase protein sequence (NCBI accession No. AAP78682) [20], through BLAST search. Recovered *Acanthamoeba* protein was further aligned with its different homologs in other organisms using MUSCLE 3.8 hosted at European Bioinformatics Institute webpage (http://www.ebi.ac.uk/Tools/msa/muscle/). The name and NCBI accession numbers of different organisms Src kinase proteins used in alignment, are *Drosophila melanogaster* c-Src (AAA28913.1), *Caenorhabditis elegans* Src1 (NP_490866), *Ephydatia fluviatilis* Src related protein (BAB83688), *Monosiga brevicollis* Src1 (EDR48627.1) *Hydra vulgaris* (AAA29217.1) and Human cSrc (NP_005408).

## Results and discussion

### *Inhibition of src kinase reduced A. castellanii growth*

**Figure 1:**
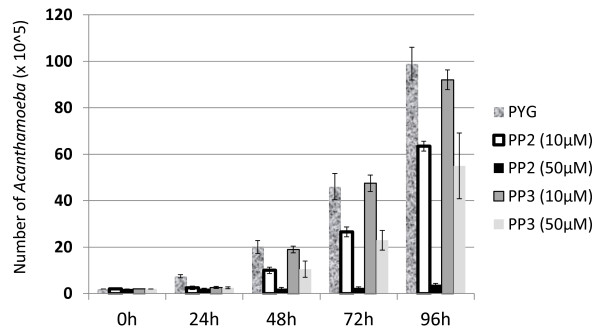
**Src kinase inhibitor, PP2 exhibited amoebistatic effects.***A. castellanii* were incubated in the presence of Src kinase-specific inhibitor, PP2 or its inactive analog, PP3 in normal growth medium (PYG) for various intervals of time. Cell numbers were determined using haemocytometer counting. Micromolar concentrations of PP2 but not PP3 abolished amoebae growth. Results represent the mean of three independent experiments performed in duplicate, bars represent standard error.

To determine the role of Src kinase on the biological properties of *A. castellanii*, growth and viability assays were performed. Growth under control conditions (amoebae incubated in growth medium in the absence of inhibitor) showed an approximate doubling of the number of cells every 24 h (Figure 1). However, the presence of Src kinase inhibitor, PP2, significantly reduced growth [*P* = 0.015 for amoebae in PYG versus amoebae in PYG plus PP2 (50 μM) at 96 h, using *T*-test, paired, one tail distribution] but did not affect amoebic viability, as determined by Trypan blue exclusion test (0% effects). At micromolar concentrations of PP2 (50 μM), amoeba growth was abolished. In contrast, inactive analog Src kinase, PP3 affected neither growth nor viability of *A. castellanii* for any time point tested [*P* = 0.051 for amoebae in PYG versus amoebae in PYG plus PP3 (50 μM) at 96 h, using *T*-test, paired, one tail distribution] (Figure 1).

### Src kinase inhibition blocked *A. castellanii* phagocytosis in a concentration-dependent manner

**Figure 2:**
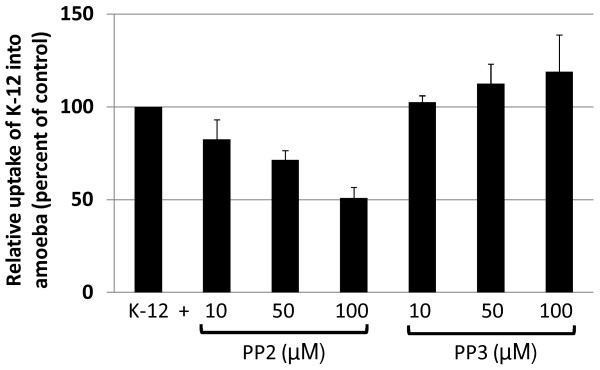
**Inhibition of Src kinase blocks*A. castellanii*phagocytosis.** The effects of Src kinase in the ability of *A. castellanii* to uptake live non-invasive *E. coli* strain, HB101. Untreated *A. castellanii* were used as 100% phagocytosis and levels of phagocytosis in inhibitor-treated *A. castellanii* are shown as percentage change. Note that Src kinase inhibitor, PP2 but not its inactive analog, PP3 exhibits a significant decrease in *A. castellanii* phagocytosis at 50 μM (*P* < 0.05). Results represent the mean of three independent experiments performed in duplicate, bars represent standard error.

To determine the effects of Src kinases on *A. castellanii* phagocytosis of *E. coli* K-12, phagocytosis assays were performed. It was observed that *A. castellanii* exhibited significant reduced bacterial uptake in the presence of PP2, specific Src kinase inhibitor [*P* = 0.038 for amoebae with K-12 versus amoebae with K-12 plus PP2 (50 μM), using *T*-test, paired, one tail distribution], although there was no difference at 10 μM (*P* = 0.21) (Figure 2). At 100 μM of PP2, more than 50% inhibition of *A. castellanii* uptake of *E. coli* K-12 strain, HB101 was observed (Figure 2). In contrast, PP3, an inactive analog of PP2, had no effect on bacterial uptake by *A. castellanii* at any of the concentrations tested (*P* = 0.127 for amoebae with K-12 versus amoebae with K-12 plus PP3 (50 μM), using *T*-test, paired, one tail distribution) (Figure 2).

### Pathogenic *E. coli* K1 invasion of A. castellanii was enhanced using Src kinase inhibitor, PP2

**Figure 3:**
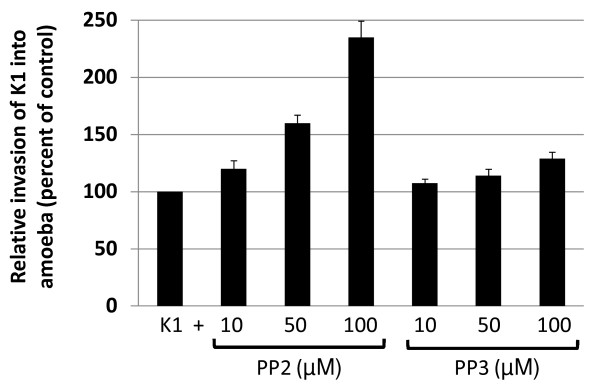
**Src kinase inhibition enhanced neuropathogenic*E. coli*K1 invasion of*A. castellanii*.** The involvement of Src kinase in the invasion of pathogenic *E. coli* (meningitis isolate) into *A. castellanii* was determined. Untreated *A. castellanii* were used as 100% phagocytosis and levels of bacterial invasion in inhibitor-treated *A. castellanii* are shown as percentage change. Src kinase inhibitor, PP2 but not its inactive analog, PP3 exhibits a significant increase in bacterial invasion of *A. castellanii* (*P* < 0.05). Results represent the mean of three independent experiments performed in duplicate, bars represent standard error of the mean.

In contrast to phagocytic uptake of non-invasive bacteria by *Acanthamoeba* (Figure 2), effects of Src kinase were reversed in the invasion of pathogenic *E. coli* K1 into *A. castellanii*. Pre-treatment of *A. castellanii* with PP2 significantly enhanced invasion of pathogenic bacteria [*P* = 0.026 for amoebae with K-12 *versus* amoebae with K-12 plus PP2 (50 μM), using *T*-test, paired, one tail distribution], although there was no difference at 10 μM (*P* = 0.077) (Figure 3). At 100 μM of PP2, invasion of a neuropathogenic *E. coli* K1 into *A. castellanii* was more than doubled (Figure 3). In contrast, PP3, an inactive analog of PP2 had no effect on bacterial uptake by *A. castellanii* at any of the concentrations tested [*P* = 0.09 for amoebae with K-12 *versus* amoebae with K-12 plus PP3 (50 μM), using *T*-test, paired, one tail distribution] (Figure 3).

### Src kinases are involved in *Acanthamoeba* protease secretions

**Figure 4:**
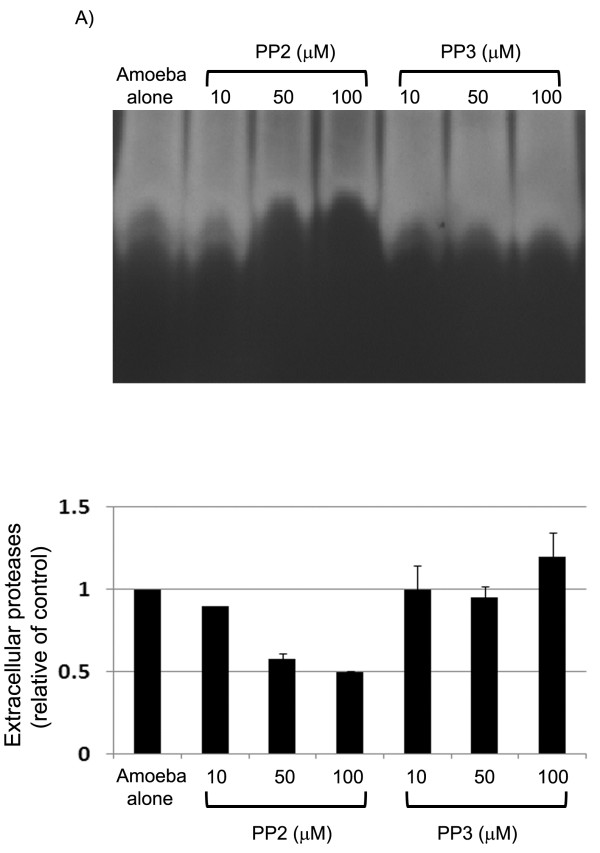
**Representative effects of Src kinase inhibitor on extracellular proteases of*Acanthamoeba*. (A)***A. castellanii* were incubated in RPMI in the presence or absence of PP2 or PP3. Conditioned medium (CM) was collected and proteolytic activities determined using zymographic assays. Note that PP2, a specific Src kinase inhibitor blocked *A. castellanii* proteases. Results are representative of three independent experiments. **(B)** The density of the bands was quantitated using an imaging densitometer. Data represent the mean of three independent experiments, bars represent standard error.

To determine the role of Src kinase in *A. castellanii* protease secretion, conditioned medium (CM) was produced in the presence of Src kinase inhibitor, PP2, or its inactive analog, PP3 and analysed for protease activities. In the absence of any inhibitor, *A. castellanii* exhibited significant proteolytic activities (Figure 4). The proteolytic activity was sensitive to phenylmethylsulfonyl fluoride (PMSF), a serine protease inhibitor, indicating specific inhibition (data not shown). In contrast, CM prepared in the presence of PP2 exhibited reduced protease activities (> 50% inhibition) (Figure 4), while PP3 exhibited no effects (Figure 4). The protease secretion was increasingly reduced with a corresponding increase in the concentration of PP2, an effect not observed when PP3 was used (Figure 4).

### Src kinases did not affect *Acanthamoeba* adhesion to and cytotoxicity of HBMEC

**Figure 5:**
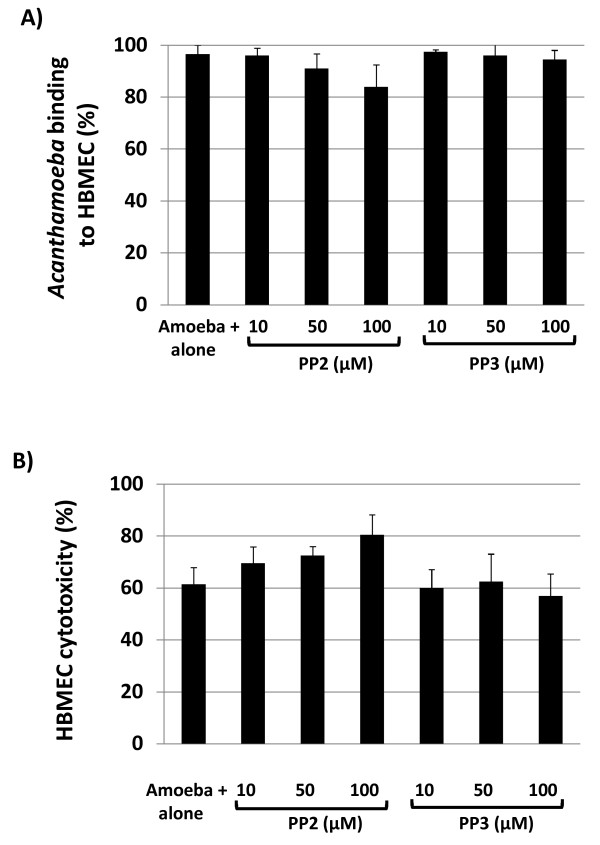
**Inhibition of Src kinase had no effect on*A. castellanii*adhesion to and cytotoxicity of HBMEC. (A)** Various concentrations of PP2 or PP3 were added to *A. castellanii* (4 x 10^5^ amoebae/well) for 30 min. After this incubation, *A. castellanii* were washed with PBS, and added to HBMEC monolayers for 1 h. The unbound amoebae were counted using a haemocytometer and the numbers of bound amoebae were deduced as follows: No. of unbound amoebae/Total number of amoebae x 100 = % unbound amoebae. The numbers of bound amoebae were deduced as follows: % unbound amoebae – 100 = % bound amoebae. **(B)** As for adhesion assays, amoebae were incubated with HBMEC for up to 24 h and the percent HBMEC cytotoxicity was determined by measuring LDH release. Results represent the mean of three independent experiments performed in duplicate. Bars represent standard error.

To determine the involvement of Src kinase in *A. castellanii* binding to and cytotoxicity of HBMEC, assays were performed. It was observed that neither PP2 nor PP3 had any significant effect on *Acanthamoeba* binding to HBMEC monolayers (Figure 5A) (At 100 μM, *P* = 0.086 for PP2 and *P* = 0.256 for PP3 respectively, using *T*-test, paired, one tail distribution). Next, to determine the effects of PP2 on *A. castellanii*-mediated HBMEC death, cytotoxicity assays were performed. In the absence of PP2, *Acanthamoeba* produced severe HBMEC cell cytotoxicity (up to 65%) within 24 h (Figure 5B). Although *A. castellanii* pre-treatment with PP2 showed increased HBMEC cytotoxicity due to amoebae, however, these effects were insignificant (At 100 μM of PP2, *P* = 0.053, using *T*-test, paired, one tail distribution) (Figure 5B). In contrast, PP3 had no effects on *A. castellanii*-mediated HBMEC cytotoxicity (At 100 μM of PP3, *P* = 0.10, using *T*-test, paired, one tail distribution) (Figure 5B).

### *Acanthamoeba* src kinase identification and sequence alignments

**Figure 6:**
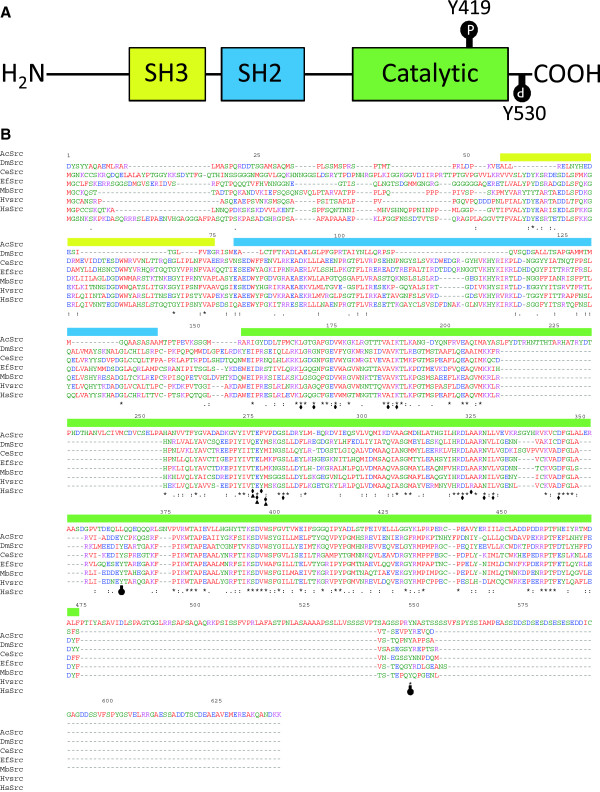
**Sequence alignment of*Acanthamoeba castellanii*Src kinase and its orthologs. (a)** Domain architecture of human c-Src kinase. The two important regulatory phosphorylation sites (Y419 and Y530) are marked. **(b)** Putative *Acanthamoeba* Src kinase (AcSrc) sequence was aligned with *Drosophila melanogaster* c-Src (DmSrc), *Caenorhabditis elegans* Src (CeSrc), *Ephydatia fluviatilis* Src related protein (EfSrc), *Monosiga brevicollis* Src1 (MbSrc), *Hydra vulgaris* (HvSrc) and *Homo sapiens* cSrc (HsSrc). The SH3, SH2 and catalytic domain are indicated with yellow, blue and green bars above the sequence, respectively. Asterisk (*), colon (:) and period (.) marks the region of conserved, strongly similar and weakly similar residues, respectively. Whereas black diamond represents residues involve in ATP binding; black arrowheads represents residues form hydrogen bond with PP2, and black circles indicates the Y419 and Y530 phosphorylation sites in human cSrc, most of which are also conserved in AcSrc.

A BLAST search of *Monosiga brevicollis* Src1 protein in the *A. castellanii* genome resulted in the identification of protein (of 639 amino acids in length), which was found to be similar in architecture to human cSrc and its orthologs (Figure 6a and b), indicated here as *Acanthamoeba castellanii* Src kinase (AcSrc). Putative AcSrc kinase shares considerable amino acid homology with its orthologs [36% with *Caenorhabditis elegans* Src1 (NP_490866); 34% with *Ephydatia fluviatilis* Src related protein (BAB83688); 33% with *Hydra vulgaris* (AAA29217.1); 31% with *Drosophila melanogaster* c-Src (AAA28913.1)]. However, it also has its unique *N*- and *C*-terminal regions which are not homologous to its orthologs but to a small hypothetical protein of the fungus *Verticillium dahliae* in NCBI database (data not shown). In the human cSrc kinases protein, phosphorylation of tyrosine at positions 419 and 530 (Y419 and Y530) marks the protein for active and inactive state, respectively [21,22]. Of these two phosphorylation sites, a tyrosine residue corresponding to Y530 of human cSrc is present in AcSrc kinase, but the tyrosine residue corresponding to Y419 of human cSrc is absent. This information suggests that the AcSrc kinase might be constitutively active in *Acanthamoeba*. The role of these conserved tyrosine residue Y530 is not clear in protist Src kinases, because the phosphorylation of MbSrc1 in *Monosiga brevicolli* at the tyrosine residue corresponding to Y530 of human cSrc kinase did not result in an inactivation of its kinase activity, unlike its human homologs [20], which may explain the functioning of AcSrc. The amino acids which take part in binding of ATP and PP2 in human Srk kinases [23,24] showed similarity in AcSrc (Figure 6b). This further supported our findings that PP2 binds and inhibits *Acanthamoeba* Src kinase, which might have a role in important cellular processes, such as growth and phagocytosis.

Recent studies have shown that *Acanthamoeba* exhibits multifactorial virulence properties to produce damage of HBMEC, which constitute the blood–brain barrier, to produce encephalitis and corneal epithelial cells to induce keratitis [4]. Here, we studied the role of Src kinases on the biology and pathogenesis of *Acanthamoeba*. The findings revealed that Src kinase inhibitor, PP2, but not its inactive analog, PP3, abolished *A. castellanii* proliferation. These findings indicate that Src kinases have a pivotal role in the growth of A. *castellanii*, which is consistent with some previous findings that showed that Src is an important signalling molecule involved in the growth of various eukaryotic cell types [11,12]. Src kinases act on a number of substrates in the cell, including enzymes involved in phospholipid metabolism, such as the p85 subunit of PI3-K, phopholipase C-γ (PLC-γ) and the signalling molecules p190RhoGAP, p120rasGAP and EGF receptor substrate, Eps8, involved in cellular transformation, proliferation and survival [25-27]. Phospholipid metabolism is important for the synthesis and maintenance of the cell membrane. Inhibition of this pathway would result in a cessation of growth and proliferation, a possible explanation for the present findings relating to the transformation of amoebae into the cyst form. The fact that Src kinases are involved in the life cycle and growth of A. *castellanii* suggest that they should be studied further.

*A. castellanii* use phagocytosis for food uptake (such as non-invasive *E. coli* K-12), whereas *E. coli* K1 (a causative agent of bacterial meningitis), utilises A. c*astellanii* as a reservoir and invades into the *Acanthamoeba*. Inhibition of Src kinase reduced A. *castellanii* phagocytosis of non-invasive K-12 with an increase in the concentration of PP2, while opposite effects were observed for invasive *E. coli* K1. A likely explanation is that *E. coli* K-12 is a food source for amoebae. In such situations, the process of bacterial uptake is most likely to be “driven” by *A. castellanii*, which would be dependent on amoeba intracellular signal transduction pathways involving Src kinases. In contrast, neuropathogenic *E. coli* K1 is an invasive strain. Their invasion of *A. castellanii* is most likely reliant on bacterial invasion, which might explain the involuntary uptake of *E. coli* K1 by *A. castellanii*, as observed in the present study. However, this contrasts with results of previous studies [16,28,29], which showed the involvement of Src in *Staphylococcus aureus*-invasion of embryonic kidney cells, *Shigella* invasion of epithelial cells and uropathogenic *E. coli*-invasion of bladder epithelial cells, which may be due to different host cell types, i.e., phagocytic *Acanthamoeba versus* non-phagocytic host cells. Future studies are needed to address these aspects in a comparative way.

*Acanthamoeba* are known to secrete large amounts of proteases that are shown to be key virulence factors in keratitis [30,31] as well as GAE infections [9,13]. For example, it is shown that serine proteases of *Acanthamoeba* increased the permeability of the blood–brain barrier by over 45% [32], while intrastromal injection of amoeba excretory products produced lesions similar to those observed in keratitis patients [31]. In the present study, it was observed that Src kinase played an important role in the protease secretion by *A. castellanii*. The proteases involved were shown to be serine proteases, inhibited by PMSF. This is significant as the inhibition of serine proteases dramatically reduce the ability of *A. castellanii* to cause inflammation and tissue damage [13,32] and possibly subsequent GAE.

Previous studies have shown that the adhesion of *A. castellanii* to the host cell is an important step in the pathogenesis of acanthamoebiasis [33,34]. Our findings revealed that Src kinase had a minimal effect on *A. castellanii* adhesion to HBMEC, which is not surprising, as Src kinases are regulators of intracellular signalling pathways, and the binding of amoebae to HBMEC is known to be mediated by mannose-binding protein expressed on the surface membranes of *Acanthamoeba*[33,34]. Given that Src kinases inhibited protease secretion and phagocytic activity of *A. castellanii* and that both properties have been implicated in the pathogenicity of *Acanthamoeba*, it was anticipated that inhibition of Src kinases would result in reduced *A. castellanii*-mediated HBMEC cytotoxicity. It was surprising that the inhibition of Src kinases had no significant effect on HBMEC cytotoxicity. However, cytotoxicity is a delayed event and PP2 effects are reversible [19]. The fact that assays were performed by pre-treatment of *A. castellanii* with PP2 for 30 min followed by their incubation with HBMEC for 24 h. in the absence of inhibitor, may explain these findings. As PP2 has toxic effects on the host cells, assays could not be performed in the presence of PP2 for longer periods of time. Future studies, using host cells expressing dominant negative forms of Src kinases, should establish their involvement in *A. castellanii*-mediated HBMEC cytotoxicity.

## Conclusions

For the first time, the present findings showed that Src kinases are involved in the proliferation, phagocytic properties and proteolytic activities of *A. castellanii*. Future studies should further explore the precise mechanisms associated with *Acanthamoeba* pathogenesis, which may help develop preventative and/or therapeutic interventions against acanthamoebiasis.

## Competing interests

The authors declare that they have no competing interests.

## Authors’ contributions

NK conceived the study. RS and MM designed and conducted all experiments under the supervision of NAK. JI performed genome analyses and interpretations. RS, JI and NAK contributed to the writing of the manuscript. All authors approved the final manuscript.

## References

[B1] Booton GC Visvesvara GS Byers TJ Kelly DJ Fuerst PA Identification and distribution ofAcanthamoebaspecies genotypes associated with nonkeratitis infections J Clin Microbiol 2005 43 1689 1693 10.1128/JCM.43.4.1689-1693.2005 15814986 PMC1081337

[B2] Corsaro D Venditti D Phylogenetic evidence for a new genotype ofAcanthamoeba(Amoebozoa, Acanthamoebida) Parasitol Res 2010 107 233 238 10.1007/s00436-010-1870-6 20411277

[B3] Nuprasert W Putaporntip C Pariyakanok L Jongwutiwes S Identification of a novel t17 genotype ofAcanthamoebafrom environmental isolates and t10 genotype causing keratitis in Thailand J Clin Microbiol 2010 48 4636 4640 10.1128/JCM.01090-10 20943863 PMC3008494

[B4] Siddiqui R Khan NA Biology and Pathogenesis ofAcanthamoeba Parasit Vectors 2012 5 6 10.1186/1756-3305-5-6 22229971 PMC3284432

[B5] Marciano-Cabral F Cabral G Acanthamoebaspp. as agents of disease in humans Clin Microbiol Rev 2003 16 273 307 10.1128/CMR.16.2.273-307.2003 12692099 PMC153146

[B6] Visvesvara GS Moura H Schuster FL Pathogenic and opportunistic free-living amoebae:Acanthamoebaspp.,Balamuthia mandrillaris,Naegleria fowleri, andSappinia diploidea FEMS Immunol Med Microbiol 2007 50 1 26 10.1111/j.1574-695X.2007.00232.x 17428307

[B7] Croft SL Seifert K Duchêne M Antiprotozoal activities of phospholipid analogues Mol Biochem Parasitol 2003 126 165 172 10.1016/S0166-6851(02)00283-9 12615315

[B8] Martinez AJ Visvesvara GS Free-living, amphizoic and opportunistic amebas Brain Pathol 1997 7 583 598 10.1111/j.1750-3639.1997.tb01076.x 9034567 PMC8098488

[B9] Khan NA Acanthamoebaand the blood–brain barrier: the breakthrough J Med Microbiol 2008 57 1051 1057 10.1099/jmm.0.2008/000976-0 18719172

[B10] Chiarugi P Src redox regulation: there is more than meets the eye Mol. Cell 2008 26 329 337 18772619

[B11] Roskoski R Src protein-tyrosine kinase structure and regulation Biochem Biophys Res Commun 2004 324 1155 1164 10.1016/j.bbrc.2004.09.171 15504335

[B12] Tatosyan AG Mizenina OA Kinases of the Src Family: structure and functions Biochem 2000 65 49 58 10702640

[B13] Khan NA Siddiqui R Acanthamoebaaffects the integrity of the human brain microvascular endothelial cells and degrades the tight junction proteins Int J Parasitol 2009 39 1611 1616 10.1016/j.ijpara.2009.06.004 19580812

[B14] Stins MF Gilles F Kim KS Selective expression of adhesion molecules on human brain microvascular endothelial cells J Neuroimmunol 1997 76 81 90 10.1016/S0165-5728(97)00036-2 9184636

[B15] Hanke JH Gardner JP Dow RL Changelian PS Brissette WH Weringer EJ Pollok BA Connelly PA Discovery of a novel, potent, and Src family-selective tyrosine kinase inhibitor. Study of Lck- and FynT-dependent T cell activation J Biol Chem 1996 271 695 701 10.1074/jbc.271.2.695 8557675

[B16] Eto DS Jones TA Sundsbak JL Mulvey MA Integrin-mediated host cell invasion by type 1-piliated uropathogenicEscherichia coli PLoS Pathog 2007 3 e100 10.1371/journal.ppat.0030100 17630833 PMC1914067

[B17] Sissons J Alsam S Stins M Rivas AO Morales JL Faull J Khan NA Use ofin vitroassays to determine effects of human serum on biological characteristics ofAcanthamoeba castellanii J Clin Microbiol 2006 44 2595 2600 10.1128/JCM.00144-06 16825391 PMC1489474

[B18] Matin A Stins M Kim KS Khan NA Balamuthia mandrillarisexhibits metalloprotease activities FEMS Immunol Med Microbiol 2006 47 83 91 10.1111/j.1574-695X.2006.00065.x 16706791

[B19] Sissons J Kim KS Stins M Jayasekera S Alsam S Khan NA Acanthamoeba castellaniiinduces host cell death via a phosphatidylinositol 3-kinase-dependent mechanism Infect Immun 2005 73 2704 2708 10.1128/IAI.73.5.2704-2708.2005 15845472 PMC1087316

[B20] Barker SC Kassel DB Weigl D Huang X Luther MA Knight WB Characterization of pp 60c-src tyrosine kinase activities using a continuous assay: autoactivation of the enzyme is an intermolecular autophosphorylation process Biochem 1995 34 14843 14851 10.1021/bi00045a027 7578094

[B21] Boggon TJ Eck MJ Structure and regulation of Src family kinases Oncogene 2004 23 7918 7927 10.1038/sj.onc.1208081 15489910

[B22] Li W Young SL King N Miller WT Signaling properties of a non-metazoan Src kinase and the evolutionary history of Src negative regulation J Biol Chem 2008 283 15491 15501 10.1074/jbc.M800002200 18390552 PMC2397478

[B23] Sicheri F Kuriyan J Structures of Src-family tyrosine kinases Curr Opin Struct Biol 1997 7 777 785 10.1016/S0959-440X(97)80146-7 9434895

[B24] Zhu X Kim JL Newcomb JR Rose PE Stover DR Toledo LM Zhao H Morgenstern KA Structural analysis of the lymphocyte-specific kinase Lck in complex with non-selective and Src family selective kinase inhibitors Structure 1999 7 651 661 10.1016/S0969-2126(99)80086-0 10404594

[B25] Kassenbrock CK Hunter S Garl P Johnson GL Anderson SM Inhibition of Src family kinases blocks epidermal growth factor (EGF)-induced activation of Akt, phosphorylation of c-Cbl, and ubiquitination of the EGF receptor J Biol Chem 2002 277 24967 24975 10.1074/jbc.M201026200 11994282

[B26] Jin W Yun C Jeong J Park Y Lee HD Kim SJ C-Src is required for tropomyosin receptor kinase C (TrkC)-induced activation of the phosphatidylinositol 3-kinase (PI3K)-AKT pathway J Biol Chem 2008 283 1391 1400 17991742 10.1074/jbc.M705052200

[B27] Windham TC Parikh NU Siwak DR Summy JM McConkey DJ Kraker AJ Gallick GE Src activation regulates anoikis in human colon tumor cell lines Oncogene 2002 21 7797 7807 10.1038/sj.onc.1205989 12420216

[B28] Agerer F Michel A Ohlsen K Hauck CR Integrin-mediated invasion ofStaphylococcus aureusinto human cells requires Src family protein-tyrosine kinases J Biol Chem 2003 278 42524 42531 10.1074/jbc.M302096200 12893831

[B29] Mounier J Popoff MR Enninga J Frame MC Sansonetti PJ Van Nhieu GT The IpaC carboxyterminal effector domain mediates Src-dependent actin polymerization duringShigellainvasion of epithelial cells PLoS Pathog 2009 5 e1000271 10.1371/journal.ppat.1000271 19165331 PMC2621354

[B30] Cao Z Jefferson DM Panjwani N Role of carbohydrate-mediated adherence in cytopathogenic mechanisms ofAcanthamoeba J Biol Chem 1998 273 15838 15845 10.1074/jbc.273.25.15838 9624184

[B31] He YG Niederkorn JY McCulley JP Stewart GL Meyer DR Silvany R Dougherty J In vivoandin vitrocollagenolytic activity ofAcanthamoeba castellanii Invest Ophthalmol Vis Sci 1990 31 2235 2240 2173683

[B32] Alsam S Sissons J Jayasekera S Khan NA Extracellular proteases ofAcanthamoeba castellanii(encephalitis isolate belonging to T1 genotype) contribute to increased permeability in anin vitromodel of the human blood–brain barrier J Infect 2005 51 150 156 10.1016/j.jinf.2004.09.001 16038767

[B33] Alsam S Kim KS Stins M Rivas AO Sissons J Khan NA Acanthamoebainteractions with human brain microvascular endothelial cells Microb Pathogen 2003 35 235 241 10.1016/j.micpath.2003.07.001 14580387

[B34] Garate M Cao Z Bateman E Panjwani N Cloning and characterization of a novel mannose-binding protein ofAcanthamoeba J Biol Chem 2004 279 29849 29856 10.1074/jbc.M402334200 15117936

